# DeepMPTB: a vaginal microbiome-based deep neural network as artificial intelligence strategy for efficient preterm birth prediction

**DOI:** 10.1186/s40364-024-00557-1

**Published:** 2024-02-14

**Authors:** Oshma Chakoory, Vincent Barra, Emmanuelle Rochette, Loïc Blanchon, Vincent Sapin, Etienne Merlin, Maguelonne Pons, Denis Gallot, Sophie Comtet-Marre, Pierre Peyret

**Affiliations:** 1 Université Clermont Auvergne, INRAE, MEDIS, F-63000 Clermont-Ferrand, France; 2 Université Clermont Auvergne, CNRS, Mines de Saint-Étienne, Clermont-Auvergne-INP, LIMOS, Clermont-Ferrand, France; 3 Department of Pediatrics, CRECHE Unit, CHU Clermont-Ferrand, Inserm CIC 1405, F-63000 Clermont-Ferrand, France; 4 Team “Translational approach to epithelial injury and repair”, Université Clermont Auvergne, CNRS, Inserm, iGReD, F-63000 Clermont-Ferrand, France; 5 Biochemistry and Molecular Genetics Department, CHU Clermont-Ferrand, 63000 Clermont- Ferrand, France; 6 Department of Obstetrics, CHU Clermont-Ferrand, F-63000 Clermont- Ferrand, France

**Keywords:** Preterm birth, Vaginal microbiome, Predictive diagnosis, Deep neural network, Artificial intelligence, Machine learning, Pregnancy, Microbial signature, Clinical data, Phenotype prediction, Model explainability.

## Abstract

In recent decades, preterm birth (PTB) has become a significant research focus in the healthcare field, as it is a leading cause of neonatal mortality worldwide. Using five independent study cohorts including 1290 vaginal samples from 561 pregnant women who delivered at term (*n* = 1029) or prematurely (*n* = 261), we analysed vaginal metagenomics data for precise microbiome structure characterization. Then, a deep neural network (DNN) was trained to predict term birth (TB) and PTB with an accuracy of 84.10% and an area under the receiver operating characteristic curve (AUROC) of 0.875 ± 0.11. During a benchmarking process, we demonstrated that our DL model outperformed seven currently used machine learning algorithms. Finally, our results indicate that overall diversity of the vaginal microbiota should be taken in account to predict PTB and not specific species. This artificial-intelligence based strategy should be highly helpful for clinicians in predicting preterm birth risk, allowing personalized assistance to address various health issues. DeepMPTB is open source and free for academic use. It is licensed under a GNU Affero General Public License 3.0 and is available at https://deepmptb.streamlit.app/. Source code is available at https://github.com/oschakoory/DeepMPTB and can be easily installed using Docker (https://www.docker.com/).

**Supplementary Information:**

The online version contains supplementary material available at 10.1186/s40364-024-00557-1.

## To the editor

Preterm birth (PTB) is a leading cause of neonatal mortality worldwide and the second most common cause of child deaths under the age of five years [1]. Additionally, premature neonates are at risk of numerous health complications, including neurological damage in early childhood but also respiratory and gastrointestinal disorders. Existing diagnostic methods involve the collection of maternal obstetric history and cervical measurements via transvaginal ultrasound imaging conducted in the first and second trimesters of pregnancy. However, diagnoses are often inaccurate, as physician experience varies and the processes can be time-consuming.

**Fig. 1:**
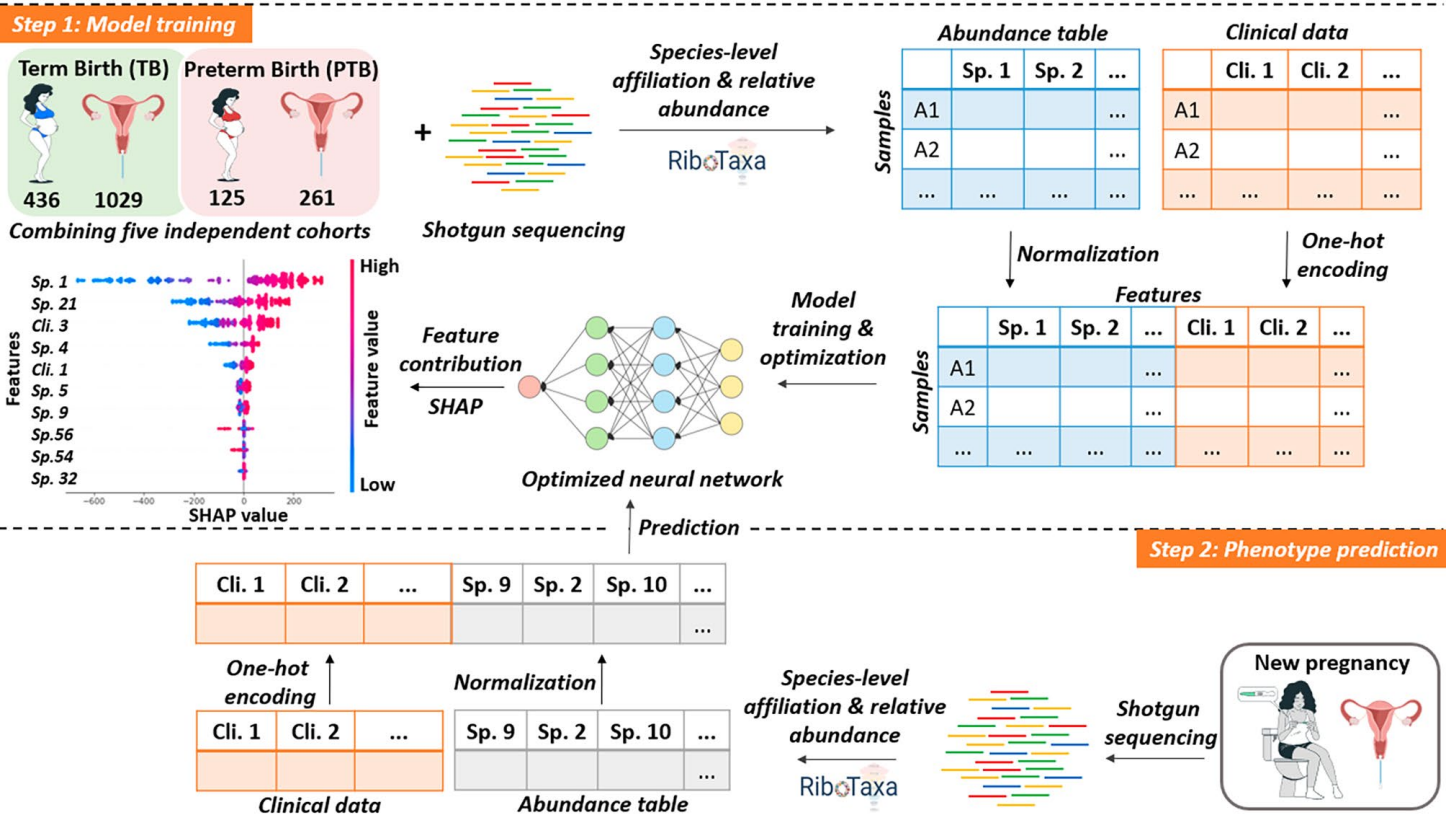
Overview of model training and phenotype prediction. For model training (step 1), the shotgun metagenomics sequences of 1290 vaginal samples from 561 pregnant women were retrieved from public databases in the form of fastq files (Table S1) [3–7]. The RiboTaxa pipeline [8] was used to obtain taxonomic profiles from the metagenomics datasets using the SILVA SSU 138.1 NR99 database. Vaginal microbiota profiles differed greatly (Welch’s *t-*test, *p* < 0.05) within individual cohorts, illustrating the heterogeneity of the vaginal population. No significant difference in the α-diversity measure was found between the TB or PTB groups. All the output taxonomy tables were grouped into a single table containing all the bacterial and eukaryotic species-level profiles of 1290 samples. In addition, the clinical data of each sample were considered. The normalized species abundances (Fig. S1) and vectorized clinical data were used to train and optimize the neural network. Features contributing to explaining the model were extracted and visualized using SHAP. To predict the phenotype based on new unknown vaginal microbiota samples (step 2), a list of features with important biomarkers contributing to the prediction was output

Existing literature suggests that vaginal microbial communities could be involved in the pathophysiology of PTB delivery [2]. This microbiome is extremely important to the host tissue, as it maintains an acidic environment, inhibits the growth of pathogenic bacteria, and modulates inflammation by cross-kingdom signalling. Despite the efforts of longitudinal studies and meta-analyses, no clear distinct microbial signatures have been characterized to identify the risk of PTB [3]. We propose DeepMPTB, a vaginal microbiota-based deep neural network (DNN) for efficient PTB prediction (Fig. 1; Supplementary Material).

**Fig. 2:**
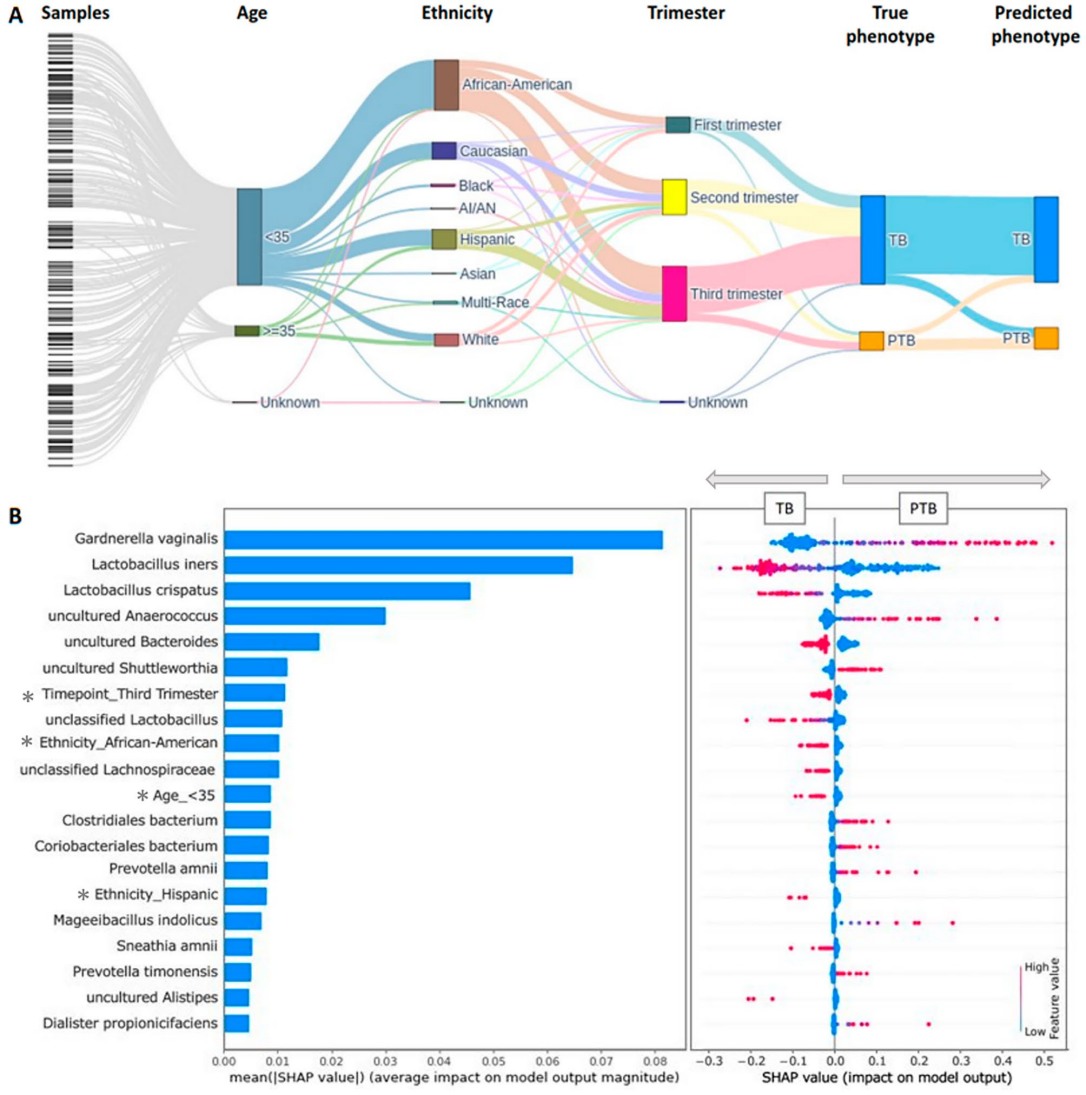
Performance of DeepMPTB based on the 20% test set (239 samples). **A** During model evaluation, the three metadata features (age, ethnicity and trimester of sample collection) were associated with each sample. For each sample, the true and predictive phenotypes were compared to evaluate the performance of DeepMPTB. **B** A summary plot for the SHAP values was generated to understand the contributions of the first 20 features in this performance analysis. Features related to clinical/demographic metadata are indicated by an asterisk. Each dot represents a sample. Negative and positive SHAP values are associated to TB and PTB prediction, respectively. Low and high SHAP values are shown in blue and red, respectively. TB: Term birth; PTB: Preterm birth

A total of 234 786 trainable parameters were optimized and the optimal hyperparameter combination for the final model (Fig. 2A, Fig. S2), included 416 units (neurons) in the 1st hidden layer and a total of 3 hidden layers, with the number of units in each layer set to half that in the preceding layer (Fig. S3). To deal with class imbalance (1029 TB and 261 PTB) in our datasets, we evaluated model performance using multiple metrics (Supplementary Material). The 20 most important features contributing to these results were also determined by the SHAP explainer (Fig. 2B). Interestingly, low-abundance species were also observed to contribute to PTB classification. Moreover, these contributing features included clinical and demographic data.

The performance of DeepMPTB was compared with that of seven state-of-the-art classification algorithms, namely, the decision tree (DT), K-nearest neighbour (KNN), random forest (RF), naïve Bayes (NB), extreme gradient boosting (XGBoost), logistic regression (LR), and support vector machine (SVM) models, which were trained and optimized based on the same input data as the DNN (Supplementary Material). DeepMPTB outperformed all other prediction models, with an AUROC score of 0.877 ± 0.11 (*p* < 0.05 for ANOVA test) and an accuracy of 84.10% (Table S2).

The model trained based on the third trimester data displayed the highest accuracy of 88%, suggesting that samples collected during the third trimester may lead to better prediction rates, although the models trained based on first and second trimester data obtained also very good accuracies of 71% and 83%, respectively (Fig. S4).

We also argue that input data quality has a significant impact on model performance (Fig. S5). We compared performance of three DNN trained using species coupled to their relative abundances determined with RiboTaxa [8] or two other popular metagenomics classifiers (*p* < 0.05 for ANOVA test) using only the biggest cohort (Supplementary Material). The DNN trained based on input data from RiboTaxa [8] showed the best performance, with an AUROC score of 0.898 ± 0.09. The DNN trained based on DeepMicrobes [9] and MetaPhlAn3 [10] data showed an AUROC score of 0.838 ± 0.14 and 0.795 ± 0.08, respectively. When only microbiome data obtained with RiboTaxa (without metadata, keeping phenotype) were used for model training, the AUROC value decreased to 0.831 ± 0.12 (*p* > 0.05 for Mann‒Whitney U test).

To show the generality of this model, we used a completely new set of 694 vaginal metagenomic data (430 TB and 264 PTB cases) from Baud et al. [11]. Overall, the optimized DNN successfully identified 80% of TB samples and 66% of the PTB samples. Importantly, phenotype prediction, especially in the case of PTB, is not determined by the presence of the same species or group of species (Fig. S6).

In conclusion, the present study presents a cutting-edge deep learning model to efficiently predict TB and PTB using vaginal microbiome data of pregnant women combined to clinical data. This new model based on data from 5 cohorts outperforms previously published machine learning-based model for PTB prediction [11, 12]. Continued accumulation of high-quality microbiome data and complete phenotypic data in perfectly controlled cohorts will certainly improve the individual phenotype prediction performance of deep learning models. Furthermore, including virome information, known to drive microbiota dynamics, would help to reach better performances. Finally, DNN enables to distinguish complex interindividual microbial interactions related to term and preterm deliveries, to highlight in-depth microorganisms potentially associated to phenotype. Interestingly we observed that different microbial profiles led to the same phenotype. This efficient TB and PTB predictive diagnosis should be highly helpful for clinicians in a personalized medicine context.

### Electronic supplementary material

Below is the link to the electronic supplementary material.

Supplementary Material 1

## Data Availability

All raw sequencing data and metadata analysed during this study were obtained from these published articles: Feehily et al. [3]. under the BioProject PRJEB34536 (61.49 Gb), Tortelli et al. [7] under the BioProject PRJNA639592 (8.52 Gb), and Goltsman et al. [5]. under the BioProject PRJNA288562 (115.53 Gb). Raw data (2.77 Tb) and metadata for Fettweis et al. [4]. cohort were received from the authors of the study following data access approval from National Institute of Health (NIH). Raw sequencing for the Pace et al. [6] cohort were available under the BioProject PRJNA451212 (15.92 Gb) and the metadata were received from the authors of the study. Supplementary material describes methods and complementary results.

## Abbreviations

AUC: Area-under-the-curve
AUROC: Area under the receiver operating characteristic curve
DL: deep learning
DNN: Deep neural network
PTB: Preterm birth
TB: Term birth

## References

[1] Liu L (2015). Global, regional, and national causes of child mortality in 2000-13, with projections to inform post-2015 priorities: an updated systematic analysis. Lancet.

[2] Menon R, Williams SM, Lamont RF (2019). Research to achieve a reduction in the global rate of preterm birth needs attention: Preface to the special issue by the preterm Birth International Collaborative (PREBIC). Placenta.

[3] Feehily C (2020). Shotgun sequencing of the vaginal microbiome reveals both a species and functional potential signature of preterm birth. NPJ Biofilms Microbiomes.

[4] Fettweis JM (2019). The vaginal microbiome and preterm birth. Nat Med.

[5] Goltsman DSA (2018). Metagenomic analysis with strain-level resolution reveals fine-scale variation in the human pregnancy microbiome. Genome Res.

[6] Pace RM (2021). Complex species and strain ecology of the vaginal microbiome from pregnancy to postpartum and association with preterm birth. Med.

[7] Tortelli BA, Lewis AL, Fay JC. *The structure and diversity of strain-level variation in vaginal bacteria*. Microb Genom, 2021. 7(3). 10.1099/mgen.0.000543 PMC8190618 33656436

[8] Chakoory O, Comtet-Marre S, Peyret P (2022). RiboTaxa: combined approaches for rRNA genes taxonomic resolution down to the species level from metagenomics data revealing novelties. NAR Genom Bioinform.

[9] Liang Q (2020). DeepMicrobes: taxonomic classification for metagenomics with deep learning. NAR Genom Bioinform.

[10] Beghini F et al. *Integrating taxonomic, functional, and strain-level profiling of diverse microbial communities with bioBakery 3*. Elife, 2021. 10. 10.7554/eLife.65088 PMC8096432 33944776

[11] Baud A (2023). Microbial diversity in the vaginal microbiota and its link to pregnancy outcomes. Sci Rep.

[12] Park S (2022). Predicting preterm birth through vaginal microbiota, cervical length, and WBC using a machine learning model. Front Microbiol.

