# Supplementary material for: DeepMPTB: a vaginal microbiome-based deep neural network as artificial intelligence strategy for efficient preterm birth prediction
Source: Biomark Res. 2024 Feb 14;12:25. doi: 10.1186/s40364-024-00557-1 (PMC10865581; doi:10.1186/s40364-024-00557-1)
Supplement: Supplementary file 1 — Supplementary Material 1 [file 40364_2024_557_MOESM1_ESM.docx]

# DeepMPTB: A vaginal microbiome-based deep neural network as artificial intelligence strategy for efficient preterm birth prediction

Oshma CHAKOORY^1^, Vincent BARRA^2^, Emmanuelle ROCHETTE^3^, Loïc BLANCHON^4^, Vincent SAPIN^4,5^, Etienne MERLIN^3^, Maguelonne PONS^3^, Denis GALLOT^4,6^, Sophie COMTET-MARRE^1^*, Pierre PEYRET^1^*

^1^Université Clermont Auvergne, INRAE, MEDIS, F-63000 CLERMONT-FERRAND, France

^2^Université Clermont Auvergne, CNRS, Mines de Saint-Étienne, Clermont-Auvergne-INP, LIMOS, Clermont-Ferrand, France.

^3^CHU Clermont-Ferrand, Department of Pediatrics, CRECHE Unit, INSERM CIC 1405, F-63000 CLERMONT-FERRAND, France.

^4^Team “Translational approach to epithelial injury and repair”, Université Clermont Auvergne, CNRS, Inserm, GReD, F-63000 CLERMONT-FERRAND, France.

^5^Biochemistry and Molecular Genetics Department, CHU Clermont-Ferrand, 63000 Clermont-Ferrand, France.

^6^Department of Obstetrics, CHU Clermont-Ferrand, F-63000 CLERMONT-FERRAND, France.

* Corresponding authors: [pierre.peyret@uca.fr](mailto:pierre.peyret@uca.fr); [sophie.marre@uca.fr](mailto:sophie.marre@uca.fr)

## Supplemental Material

### Study design

To train the deep neural network (DNN), we used existing sequencing data generated by studies focusing on vaginal microbiota associated to TB versus PTB. We focused on studies that used shotgun metagenomics sequencing to avoid PCR biases and to assess the microbiota at high resolution in order to enhance identification of microbial signatures associated with PTB and TB. Retrieved metagenomics datasets were reanalysed using the pipeline RiboTaxa, based on the reconstruction of the full-length rRNA gene to obtain a species-level identification of vaginal microbiota along relative abundance estimation of identified species. An advantage of this strategy was also to enable identification of new species as well as rare microorganisms that will not be detected using gene catalogues. Furthermore, reanalysis of sequencing data ensures uniformity of microbial data and is well suited for downstream deep learning analyses, enabling the development of a single model that can use data collected under distinct study. The controlled and high-quality species level relative abundance profiles and three clinical data were used to train and validate a deep neural network to predict the risk of PTB.

### Data collection

A systematic and comprehensive review of the literature was performed on PubMed and Google Scholar on January 20, 2021, by two independent researchers using the search terms “vaginal microbiome” AND “shotgun metagenomics” AND “preterm birth”. A total of 11 articles were obtained that were carefully screened by manually checking the sequencing methodology to select studies that applied shotgun metagenomics using the Illumina platform and exclude studies that applied only 16S rRNA gene metabarcoding. Additionally, to be included in this meta-analysis, the studies needed to report pregnant women who went on to deliver either at term or preterm and the following metadata: ethnicity and age of the participants and the week/trimester of sample collection.

### Data availability

Raw data and metadata for the Feehily *et al.* [1] cohort were downloaded from the European Nucleotide Archive (ENA) under BioProject PRJEB34536 (61.49 Gb). Raw data and metadata for the Tortelli *et al.* [2] cohort were downloaded from the Sequence Read Archive (SRA) under BioProject PRJNA639592 (8.52 Gb). Raw data and metadata for the Goltsman *et al.* [3] cohort were downloaded from the Sequence Read Archive (SRA) under BioProject PRJNA288562 (115.53 Gb). Raw data (2.77 Tb) and metadata for Fettweis *et al.* [4] cohort were received from the authors of the study following data approval from the National Institutes of Health (NIH). Raw data and metadata for the Pace *et al.* [5] cohort were downloaded from the SRA under BioProject PRJNA451212 (15.92 Gb), and the metadata were acquired from the authors of the study.

### Definition of selected cohorts for DNN training

Selected cohorts refer to prospective studies, indicating that vaginal microbiota were sampled during pregnancy and were selected after premature or at term delivery. PTB refers to those women who delivered prior to 37 weeks’ gestation. TB refers to women who delivered full term (≥ 37 weeks gestation) except in Fettweis et al. [4], where full term was considered at ≥39 weeks gestation. Most of studies indicated clearly that spontaneous PTB was the PTB phenotype targeted, except for Pace et al. [5], for which no information was available.

For each cohort, we retained the following sample metadata: phenotypes (term birth-TB, preterm birth-PTB), timepoints of sample collection (1^st^ trimester: 1-13 weeks, 2^nd^ trimester: 14-26 weeks, 3^rd^ trimester: >=27 weeks), age of participants (<35 years old, >=35 years old), ethnic group (African-American, American-Indian/American-Native - AI/AN, Asian, Black, Caucasian, Hispanic, Multi-Race, White) and participant ID. A total of 1298 samples were recovered, among which 8 samples were discarded because of a lack of phenotype descriptions (Table S1). However, few samples with unknown metadata descriptions (except phenotype) were kept for model training to enable the model to handle metadata missing values that may occur during clinical data collection.

**Table S1** General properties of individual studies included in this study: vaginal microbiome of five cohorts with term- and preterm-delivering participants. TB: Term birth; PTB: Preterm birth.

|  | Feehily *et al.* [1] | Fettweis *et al.* [4] | Goltsman *et al.* [3] | Pace *et al.* [5] | Tortelli *et al.* [2] | Total |
| --- | --- | --- | --- | --- | --- | --- |
| Number of participants | 49 | 231 | 10 | 78 | 193 | 561 |
| Number of samples | 49 | 832 | 96 | 118 | 195 | 1290 |
| Number of preterm samples | 8 | 157 | 36 | 19 | 41 | 261 |
| Overall preterm sample ratio | 16% | 18% | 38% | 16% | 21% | - |
| **Statistics on trimester of sample collection** | | | | | | |
| Number of samples from trimester 1 | - | 86 TB  28 PTB | 11 TB  12 PTB | - | 42 TB  13 PTB | 139 TB  53 PTB |
| Number of samples from trimester 2 | 41 TB  8 PTB | 200 TB  60 PTB | 22 TB  15 PTB | 10 TB  10 PTB | 41 TB  11 PTB | 314 TB  104 PTB |
| Number of samples from trimester 3 | - | 386 TB  67 PTB | 27 TB  9 PTB | 89 TB  9 PTB | 71 TB  17 PTB | 573 TB  102 PTB |
| Number of samples from unknown trimester | - | 3 TB  2 PTB | - | - | - | 3 TB  2 PTB |
| **Statistics of ethnic groups of participants** | | | | | | |
| Number of samples from African American individuals | - | 441 TB  102 PTB | - | 11 TB  2 PTB | - | 452 TB  104 PTB |
| Number of samples from Hispanic individuals | - | 20 TB  12 PTB | 10 TB  9 PTB | 81 TB  17 PTB | 45 TB  11 PTB | 156 TB  49 PTB |
| Number of samples from Caucasian individuals | 2 TB  1 PTB | 172 TB  17 PTB | - | 2 TB  0 PTB | - | 176 TB  18 PTB |
| Number of samples from White individuals | 36 TB  7 PTB | - | 30 TB  22 PTB | - | 78 TB  18 PTB | 144 TB  47 PTB |
| Number of samples from Black individuals | 2 TB  0 PTB | - | - | - | 16 TB  6 PTB | 18 TB  6 PTB |
| Number of samples from individuals with multiple races | - | 30 TB  11 PTB | 9 TB  5 PTB | - | 1 TB  2 PTB | 40 TB  18 PTB |
| Number of samples from Asian individuals | 1 TB  0 PTB | 3 TB  3 PTB | 11 TB  0 PTB | 5 TB  0 PTB | 12 TB  2 PTB | 32 TB  5 PTB |
| Number of samples from AI/AN individuals | - | 0 TB  5 PTB | - | - | 0 TB  2 PTB | 0 TB  7 PTB |
| Number of samples from individuals with unknown ethnicities | - | 9 TB  7 PTB | - | - | 2 TB  0 PTB | 11 TB  7 PTB |
| **Statistics on age of participants** | | | | | | |
| Number of samples from individuals <35 years old | 28 TB  6 PTB | 640 TB  149 PTB | 40 TB  36 PTB | 71 TB  15 PTB | 128 TB  33 PTB | 907 TB  239 PTB |
| Number of samples from individuals >=35 years old | 13 TB  2 PTB | 25 TB  8 PTB | 20 TB  0 PTB | 28 TB  4 PTB | 26 TB  8 PTB | 112 TB  22 PTB |
| Number of samples from individuals with unknown ages | - | 10 TB  0 PTB | - | - | - | 10 TB  0 PTB |

### Sequencing data preprocessing using RiboTaxa

Microbiota diversity analysis was focused on bacterial and eukaryotic diversity through the reconstruction of the full to near full-length SSU rRNA gene sequences (16S and 18S rRNA) [7], therefore excluding virome analysis, to reach the species level identification. For this, the RiboTaxa pipeline [6] was used to obtain taxonomic profiles from raw metagenomics datasets through using the SILVA SSU 138.1 NR99 database. For each cohort, raw shotgun reads were provided as input to RiboTaxa. The sequences were processed to remove Illumina adapters and known Illumina artefacts and to quality-trim both ends to Q20. The resulting reads containing more than one ‘N’, quality scores averaging less than 20 over the read, or lengths less than 60 bp after trimming were discarded. For SSU rRNA gene reconstruction, the default parameters were used except for the following parameters (Feehily cohort: --max_read_length = 300, --insert_mean =120, --insert_stddev = 300; Fettweis cohort: --max_read_length = 301, --insert_mean =120, --insert_stddev = 300; Goltsman cohort: --max_read_length = 151, --insert_mean =146, --insert_stddev = 100; Pace cohort: --max_read_length = 151, --insert_mean =100, --insert_stddev = 142; Tortelli cohort: --max_read_length = 75, --insert_mean = 75, --insert_stddev = 50), which were determined according to the sequencing length of the input datasets. For each cohort, the parameter --max_read_length represented the longest read size in the input dataset, and --insert_mean and –insert_stddev were estimated using mean_size.py (<https://gist.github.com/timoast/af73c0e9fac00187ee49>). The reconstructed SSU sequences were then classified at different taxonomic levels, from domain to species, and after discarding human eukaryotic sequences (18S rDNA), the relative abundances were calculated by RiboTaxa. The α-diversity of the samples was measured by the Chao 1 and Shannon diversity indices.

The output taxonomy tables were grouped into a single table containing all the species-level profiles using RiboTaxa_group_taxonomy.sh in RiboTaxa and used as input to the neural networks. No feature pre-selection has been made. As a result, all identified species (636 species) were used for model training to keep the overall diversity and individuality of microbiota.

### Data preparation, vectorization and normalization

653 features including 636 species and their relative abundance, and 17 clinical data features (9 ethnic groups including “unknown” for some samples, 3 age groups including “unknown” for some samples and 4 timepoints including “unknown” for some samples).

The species abundance profiles and clinical information including ethnicity, age, phenotype and timepoint of sample collection were the primary input to the deep neural network (DNN) (Fig. 1). Before fitting the data in the DNN model, a one-hot encoding method was applied to all categorical features (ethnicity, age, phenotype and timepoint of sample collection). Converting categorical features to binary vectors (0 or 1) is a required preprocessing step, as most machine learning (ML) algorithms cannot work directly with these data. Hence, we transformed all the categorical features into numerical forms using LabelEncoder in the scikit-learn[8] library, i.e., all the elements in the vector were converted to 0 except the categorical variable, which was converted to 1. After applying the one-hot encoding technique to all the categorical features, we obtained a dataset of 17 categorical features, with 9 ethnic groups including “unknown” for some samples, 3 age groups including “unknown” for some samples and 4 timepoints including “unknown” for some samples.

The variability in library sizes and sequencing depth among the independent cohorts leads to strong dependencies among the abundances of the different taxa. Hence, the species abundances were normalized to a common range to ensure that greater feature values (more abundant taxa) do not dominate over smaller feature values (less abundant taxa) [9] while preserving data comparability across microbiome samples or groups of samples. Therefore, we applied a standard scale to normalize each feature through the following transformation, called min–max normalization on a per-dataset (combined 5 cohorts) basis (Fig. S1):

$$x^{'}=\frac{x-x_{min}}{x_{max}-x_{min}}$$

Where $x$is the original data, $x^{'}$ is the normalized data, and $x_{min}, x_{max}$ are the maximum and minimum values of the original data, respectively.

The above equation is a linear transformation that maintains all the abundance ratios of the original data after normalization.


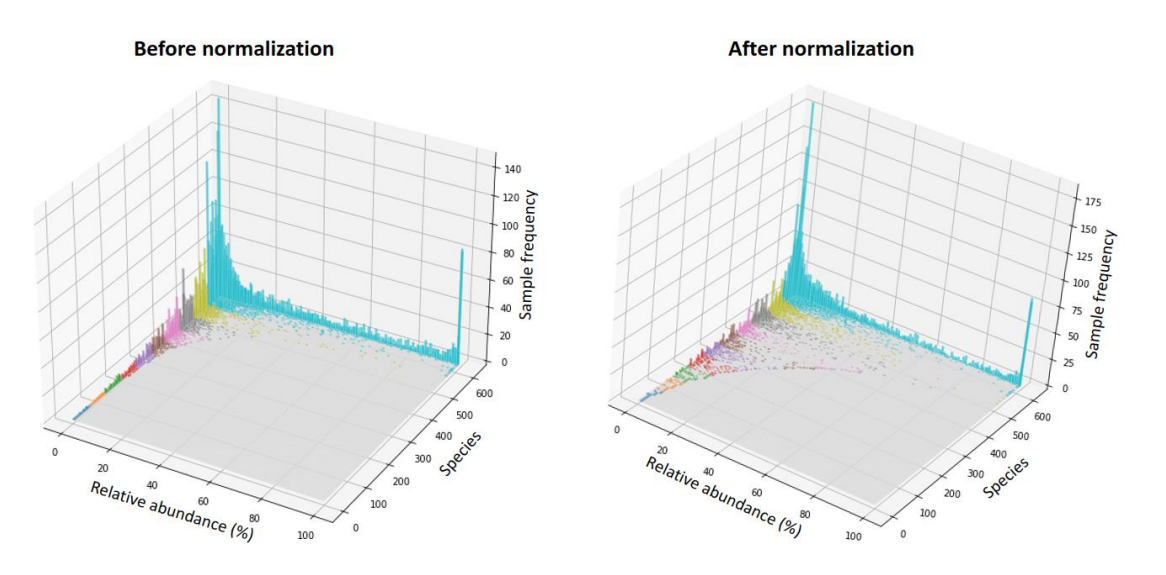


**Fig. S1**. Min–max scale transformation of species abundances obtained from the 5 cohorts. Only nonzero values are shown before and after normalization.

### Deep neural network architecture

We implemented a fully connected DNN model using the Python programming language and dedicated libraries (scikit-learn [8], TensorFlow [10] and Keras [11]).

We employed the rectified linear unit (ReLU) [12] activation function for all hidden layers. Activation functions are important for training neural networks. They provide the necessary nonlinearity to ensure that the model can learn complex representations [13]. We applied the dropout (0.1-0.5) technique to each hidden layer, which is a simple method to prevent overfitting in neural networks [14–16]. Dropout is a learning method that involves the random removal of hidden layer nodes during training, with the deleted nodes being excluded from the subsequent steps. The activation function of the output layer used the Softmax [17] function to assign a value between 0 and 1 to each class, which can be interpreted as a probability. We used the cross-entropy between the target value and the predicted value as the loss function, and we optimized this loss during each epoch using the Adam optimizer [18] with varying learning rates, with a minimum starting learning rate of 0.0001 and a maximum learning rate of 0.01. The number of nodes in each hidden layer and the number of hidden layers were considered in the design of the model. We varied the number of hidden layers (1 to 6 with better results using 3), the number of epochs (1 to 40), and the number of units in the first hidden layer (32 to 512 in steps of 32). The number of units in the hidden layers was set to half of that in the preceding layer except the first hidden layer, that helps the learning algorithm to converge faster. In our experiments, we implemented these optimizations using the ‘randomsearch’ algorithm of Keras tuner (<https://github.com/keras-team/keras-tuner>) and optimized a total of 234 786 trainable model parameters (weight/bias). The optimal model was selected based on a low validation set loss to select the best hyperparameters for the DNN. Training was stopped if the validation set loss no longer improved after 10 epochs.

### Model training

For model training, we divided a total of 1290 samples into training and test sets at a ratio of 8:2, with a given random partition seed, keeping the ratio between classes (TB and PTB) in both the training and test sets as the same as that in the given dataset. Using only the training set, learning models were trained, during which 10-fold cross-validation was applied with the training set by varying hyperparameters. Our aim was to build and fine-tune a model that was trained based on different subsets of the training data. This approach not only allowed more efficient usage of the available training data but also increased the reliability of the prediction models [19]. We used nine-folds of the training set to train the learning models and the remaining one-fold to validate the models (Fig. S2).


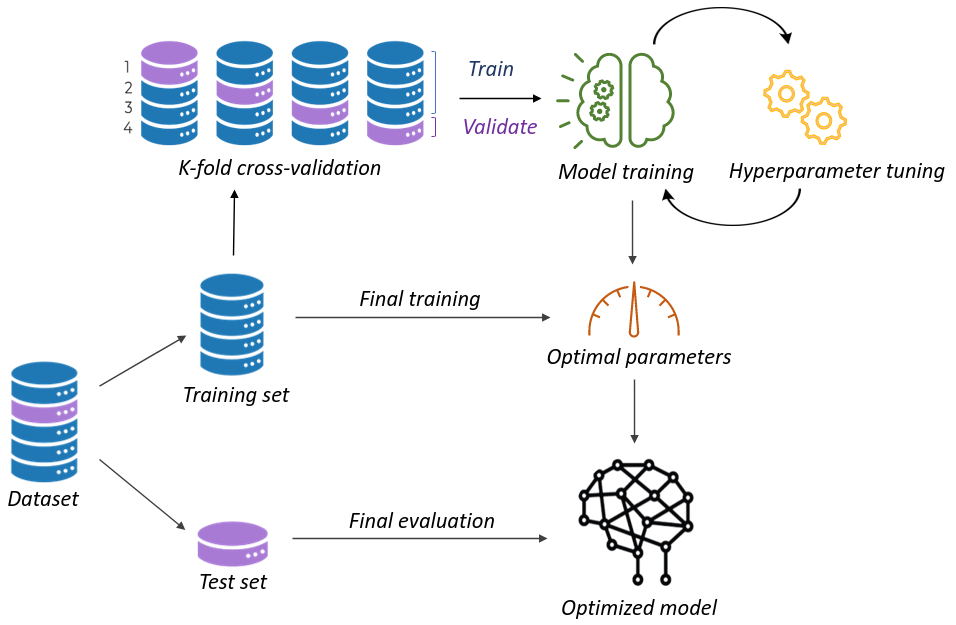


**Fig. S2** Model training and hyperparameter tuning. For model training, the initial dataset was divided at a ratio of 8:2, with 80% of the data in the training set and the remaining 20% of the data in the test set. Using the training set, cross-validation with ten-folds was applied during training. Nine-folds of the training set were used to train the learning models, and the remaining one-fold was used to validate the models. Each model was built based on different subsets of the training data and was tuned by varying the hyperparameters.

The best hyperparameter combination for each model was selected by averaging the accuracy metric of the ten different results. Once the best combination of hyperparameters was determined, we trained a final classification model using the whole training set and tested the model based on the test set (Fig. S3).


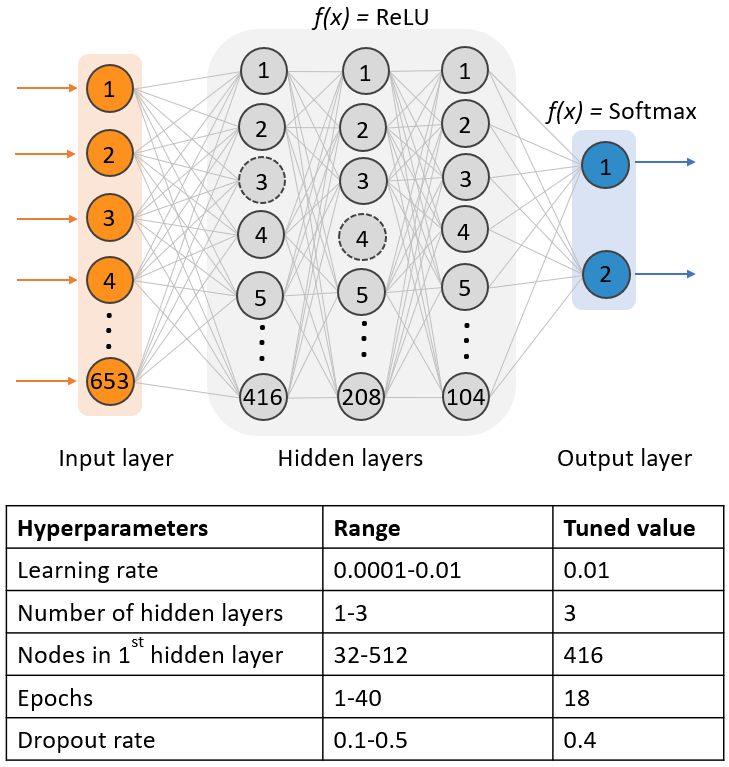


**Fig. S3** Final architecture of DeepMPTB (size of 275 kb). Five hyperparameters were tuned using Keras tuner under the ranges indicated in the bottom table. The number of nodes in the input layer represents the number of input features (653 input features). The optimized number of nodes in the first hidden layer is 416 nodes, which is successively halved in the next 2 hidden layers. The output layer consists of 2 nodes representing the two classes (TB and PTB). The ReLU and softmax activation functions (*f(x)*) were applied to the hidden and output layers, respectively. The dotted nodes within the hidden layers represent the nodes that were randomly switched off using the dropout method.

### Benchmark comparison of classification algorithms

The performance of the optimized DNN was compared with that of seven state-of-the-art classification algorithms, namely, the decision tree (DT) [20], K-nearest neighbour (KNN) [21], random forest (RF) [22], naïve Bayes (NB) [23], extreme gradient boosting (XGBoost) [24], logistic regression (LR) [25], and support vector machine (SVM) [26] models. All these models were implemented in Python (version 3.9.10). For the RF, KNN, NB, DT, LR, and SVM models, the scikit-learn [8] library (v0.24.2) was used. For XGBoost, the XGBoost [24] library (v1.5.0-dev) was used. Each model was trained based on the meta-analysis of 1290 samples to identify the best hyperparameters and configurations using the grid search cross-validation (GSCV) method in scikit-learn. The GSCV method identifies the best combination of hyperparameters during the 10-fold cross-validation process to obtain the optimal model performance [27]. The ANOVA test was used to determine significant changes in the AUROC between DeepMPTB and different ML models; a *p* value was assessed as significant when <0.05.

**Decision tree (DT):** The decision tree model was fitted with the following hyperparameter values: maximum depth = [2, 3, 4, 5, 6, 7, 8, 9, 10], maximum number of leaf nodes = [8, 18, 28, 38, 48, 59, 69, 79, 89, 100], node splitting criterion = [‘gini’, ‘entropy’], class_weight = ['balanced', none], and number of cross-validations = 3.

**K-nearest neighbour (KNN):** The KNN model was optimized with the following hyperparameter values: k = [1, 2, …, 21], metric = ['Euclidean', Chebyshev, 'Manhattan'], abd weights = ['uniform', 'distance'].

**Random forest (RF):** The RF model was optimized with the following hyperparameter values: n_estimators = [10, 100, 500, 1000]. The other parameters were the same as those for the DT model.

**Naïve Bayes (NB):** We optimized the naïve Bayes classifier with the following hyperparameter values: alpha = [1, 2, …, 500].

**Extreme gradient boosting (XGBoost):** The XGBoost model was optimized using the following hyperparameter values: gamma = [0, 0.01, 0.1, 0.3, 0.5, 0.9], max_depth = [3, 4, 5, …, 30, 31], and learning_rate = [0, 0.01, 0.1, 0.3, 0.5].

**Logistic regression (LR):** The LR model was optimized using the following hyperparameter values: solver = ['newton_cg', 'lbfgs', 'sag', 'saga'], penalty = ['none', 'l1', 'l2', 'elasticnet'], and c = [100, 10, 1.0, 0.1, 0.01].

**Support vector machine (SVM):** The SVM model was optimized with the following hyperparameter values: c = [2^−5^, 2^−3^… 2^15^] and kernels = ['linear', 'poly', 'rbf', 'sigmoid']. The default degree (for poly kernel = 3) and gamma coefficient ('scale') parameters were used.

**Table S2** Performance comparison of the predictive models

| **Models** | **Input size (n)** | **Accuracy (%)** | **AUROC** |
| --- | --- | --- | --- |
| DNN (DeepMPTB) | 1290 | 84.10 | 0.875±0.11 |
| LR | 1290 | 59.12 | 0.620±0.23 |
| KNN | 1290 | 58.06 | 0.592±0.15 |
| SVM | 1290 | 57.86 | 0.591±0.19 |
| DT | 1290 | 55.25 | 0.566±0.14 |
| RT | 1290 | 50.76 | 0.517±0.20 |
| XGBoost | 1290 | 48.72 | 0.493±0.24 |
| NB classifier | 1290 | 40.13 | 0.437±0.26 |

### Comparing performances of DNNs trained with data subsets

Among the clinical data features, the impact of the trimester of sample collection was evaluated. We trained three DNNs based on individual trimester data (first, second and third trimesters). Performances of each DNN was evaluated to determine the trimester that will lead to the best prediction performance (Fig. S4). We also evaluated the performance of a DNN trained with all samples but without demographic and clinical metadata (keeping only phenotype and microbiome data) to evaluate importance of metadata in the training of the DNN.


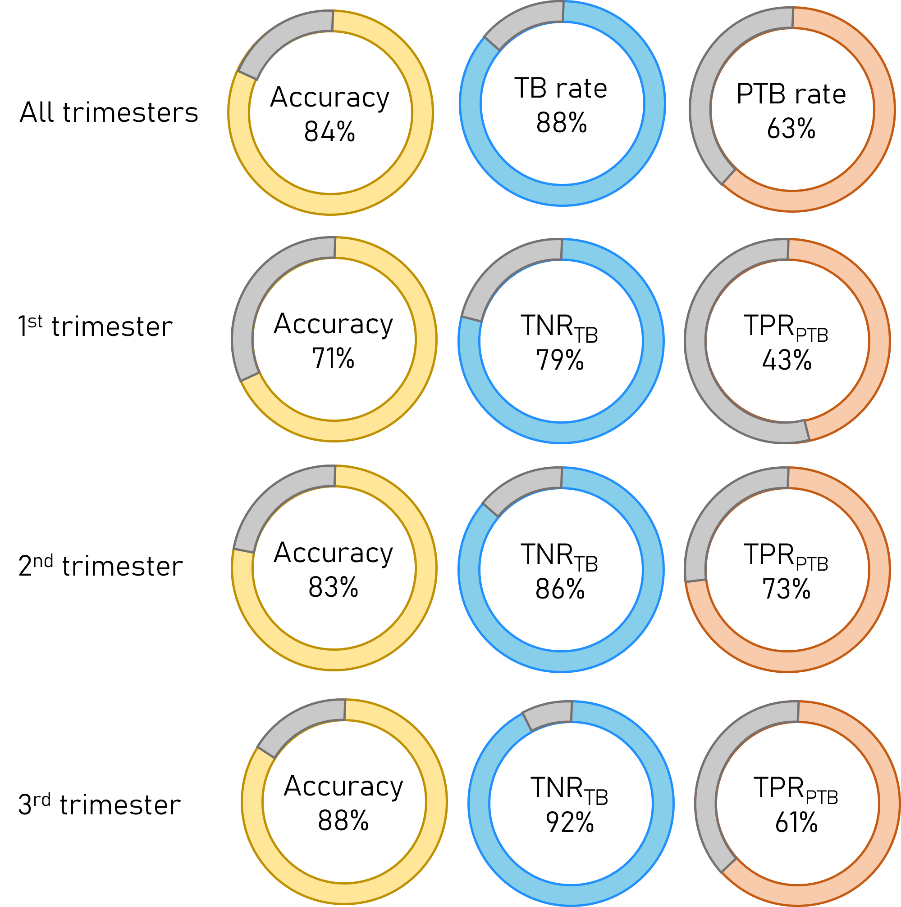


### **Fig. S4**. Performance measures of DNNs trained on data from each trimester.

### Comparing the effect of the input data on model training

Good-quality data are crucial to achieve high-performing models. In our study, the input data consisted of microbial abundance profiles obtained from RiboTaxa,[6] which is based on the reconstruction of SSU rRNA genes prior to taxonomic classification. We compared the model training accuracy with taxonomic and abundance profiles of 832 metagenomic samples in the Fettweis cohort provided by RiboTaxa and two other metagenomics classifiers: MetaPhlAn3 and DeepMicrobes (Fig. S5).

MetaPhlAn3 [28,29] uses clade-specific marker genes to rapidly assess the presence and abundance of taxa in complex metagenomes. MetaPhlAn3 was applied using the default parameters based on the database CHOCOPhlAnSGB (vJan21). Only species abundance profiles were extracted from the output of MetaPhlAn3 and used as input data for DNN training.

DeepMicrobes [30] (v1.0.0) is a computational framework based on deep learning techniques for taxonomic classification of shotgun metagenomics data. Prior to microbial classification, DeepMicrobes was trained based on the SILVA [7] SSU 138.1 NR99 database, the same database used for RiboTaxa. The database was first filtered to exclude sequences not assigned at the species level. The final species collection included 205 384 sequences. Each sequence was split into individual files using seqkit split and labelled using fna_label.py (<https://github.com/MicrobeLab/DeepMicrobes>). Next, the ART [31] Illumina read simulator was used to equalize the proportion of 250 bp reads using the MiSeq v3 2500 error model (MSv3), paired-end reads with an insert size of 300 bp and a standard deviation of 10 bp. The ART simulator was used to perform automatic sampling of both forward and reverse reads. During the training phase, a pair of reads was considered as two distinct single-end reads. Following the simulation process, a random trimming of reads from the 3' end was conducted, with an equal probability of 125-250 bp [30]. A numerical label was assigned to each read according to the species from which it was simulated. Prior to training, the reads and their respective labels were shuffled and subsequently transformed into the TensorFlow format TFRecord, which is a binary format that can be readily utilized as input for the model. The TFRecord files were used as input for model training using the default settings to obtain a species-trained model. Microbial profiling was performed using the species model [30]. Paired-end files of each sample were interleaved and converted to the TensorFlow format TFRecord using tfrec_predict_kmer.sh (from DeepMicrobes). Read classification and abundance calculation were performed using predict_DeepMicrobes.sh and report_profile.sh (from DeepMicrobes), respectively.

All taxonomic files were concatenated into a single file and used to train the DNN model. To compare RiboTaxa with MetaPhlAn3 and DeepMicrobes, we trained another DNN using the RiboTaxa results of only the Fettweis cohort. Each DNN model was optimized and trained using the methods described above. The ANOVA test was used to determine significant changes in the AUROC between the model trained on input data from different classifiers; a *p* value was assessed as significant when <0.05.


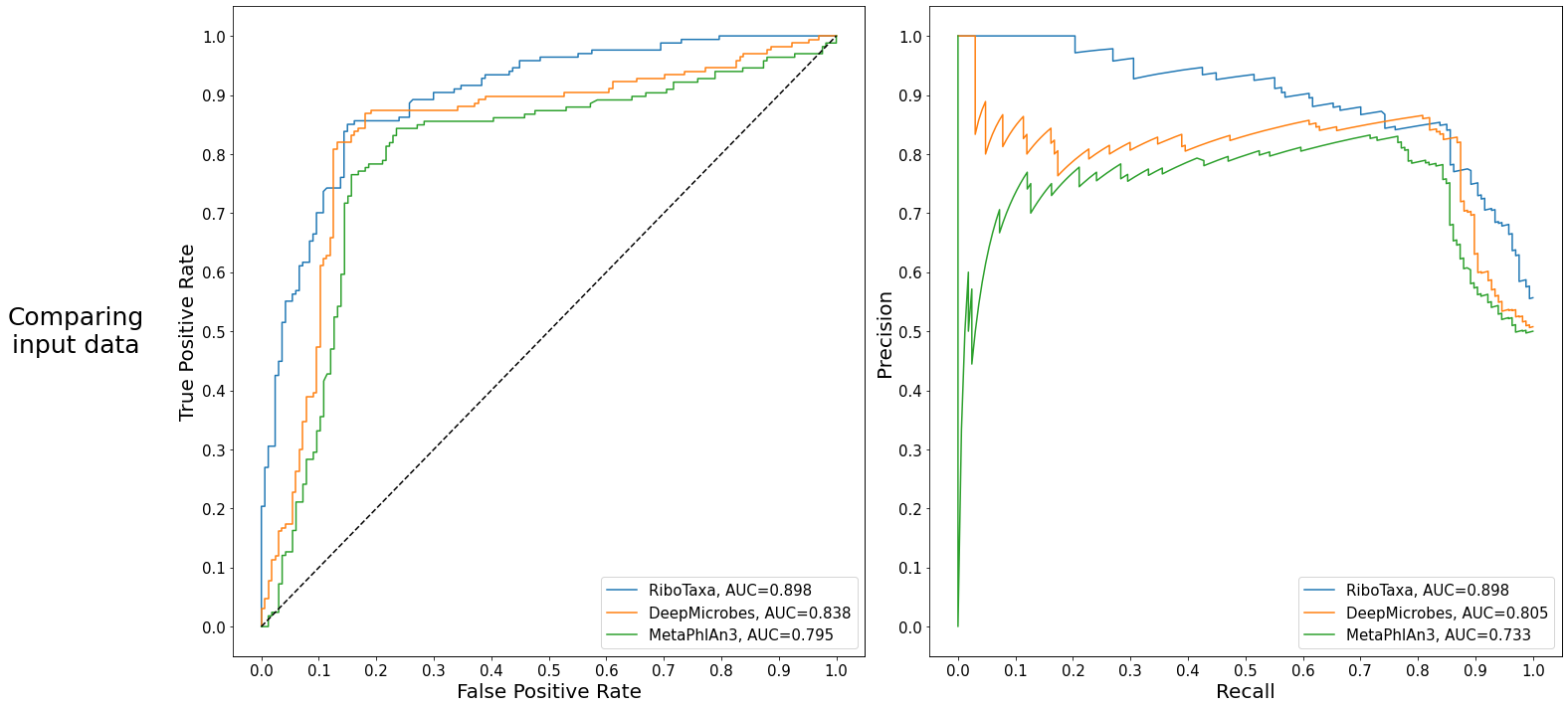


**Fig. S5** Receiver operator characteristic (ROC) (left) and precision-recall (PR) (right) curves based on the Fettweis cohort (832 samples). Three DNNs were optimized using input data from three different algorithms: (i) RiboTaxa based on the reconstruction of rRNA genes, (ii) the MetaPhlAn3-based marker gene database to perform taxonomic classification based on raw metagenomic samples, and (iii) DeepMicrobes, a deep neural network to classify metagenomics sequences directly at the species levels. AUC: area under the curve.

### Performance evaluation

The model performance based on the test set was evaluated by comparing the true sample phenotype with the predicted sample phenotype. If the model correctly classified the PTB sample, it is considered to be a true positive (TP); otherwise, it is a false positive (FP). On the other hand, if the model correctly classifies a TB sample, it is considered to be a true negative (TN); otherwise, it is a false negative (FN). We applied multiple evaluation metrics due to class imbalances to assess model performance using accuracy, true positive rate (TPR) and true negative rate (TNR), the area under the curve (AUC) of the receiver operating characteristic (ROC)/AUROC and precision-recall AUC (PR-AUC).

The accuracy is the ratio of the number of true predictions to the total number of predictions and is calculated as follows:

$$Accuracy =\frac{TP+TN}{TP+FP+TN+FN}$$

The TPR, also called sensitivity or recall is the ratio of correctly classified PTB samples (TPR_PTB_) to the total number of predicted PTB samples and is calculated as follows:

$${TPR}_{PTB} =\frac{TP}{TP+FN}$$

The TNR, also called specificity is the ratio of correctly classified TB samples (TNR_TB_) to the total number of predicted TB samples and is calculated as follows:

$${TNR}_{TB} =\frac{TN}{TN+FP}$$

Finally, the AUROC compares the false positive rate (FPR) over the TPR[32] and PR-AUC measures the recall over the precision (ratio of TP to the total number of TP and FP). AUCs were calculated using the scikit-learn [8] package and plotted using matplotlib [33] (v3.1). ROC curves and Sankey plots were generated using matplotlib [33] and plotly [34] (v5.15.0), respectively, for model comparison and to evaluate the final performance of the model based on the test set. The 95% CIs of the AUCs were estimated using the bootstrap[35] method with 1000 iterations. The Mann‒Whitney U test [36] was used to determine significant changes in the AUROC and PR-AUC between the model trained with metadata and microbiome data and without metadata (microbiome data only); a *p* value was assessed as significant when <0.05.

### SHAP

The models were interpreted by calculating feature importance scores, which describe how important the feature is for model classification [37]. In our study, we assessed feature importance using the SHAP value to explain features’ contributions. The function DeepLIFT [38,39] also known as SHAP’s DeepExplainer decomposes the output prediction of a neural network with respect to a particular input through the backpropagation of the contributions made by all neurons in the network to each feature of the input.

### Phenotype prediction on unseen data

We identified a new cohort from Baud et al., [40] that included 694 vaginal samples (430 TB and 264 PTB cases) collected from 694 mothers at the time of delivery only. The cohort included mothers with spontaneous delivery as well as with premature rupture membrane. This cohort was not used during training, and we used data from this cohort to evaluate the trained DNN model on new samples. Raw shotgun metagenomics data for Baud et al. (2023) were downloaded from ENA under the BioProject PRJEB59811 (261.26 Gb). Species level classification was performed using RiboTaxa, and after discarding non-bacterial taxa, a total of 694 bacterial species remained. Since information about ethnicity and age was not provided by the authors, they were replaced by ‘unknown’ values. Prior to prediction, the species abundances were normalized by min–max scaling, and the metadata were encoded using one-hot encoding. An individual abundance table including clinical data was input into the trained model, and the prediction value was recorded. Ultimately, each prediction was compared with the true predicted value to determine the number of correct predictions. SHAP plots for each
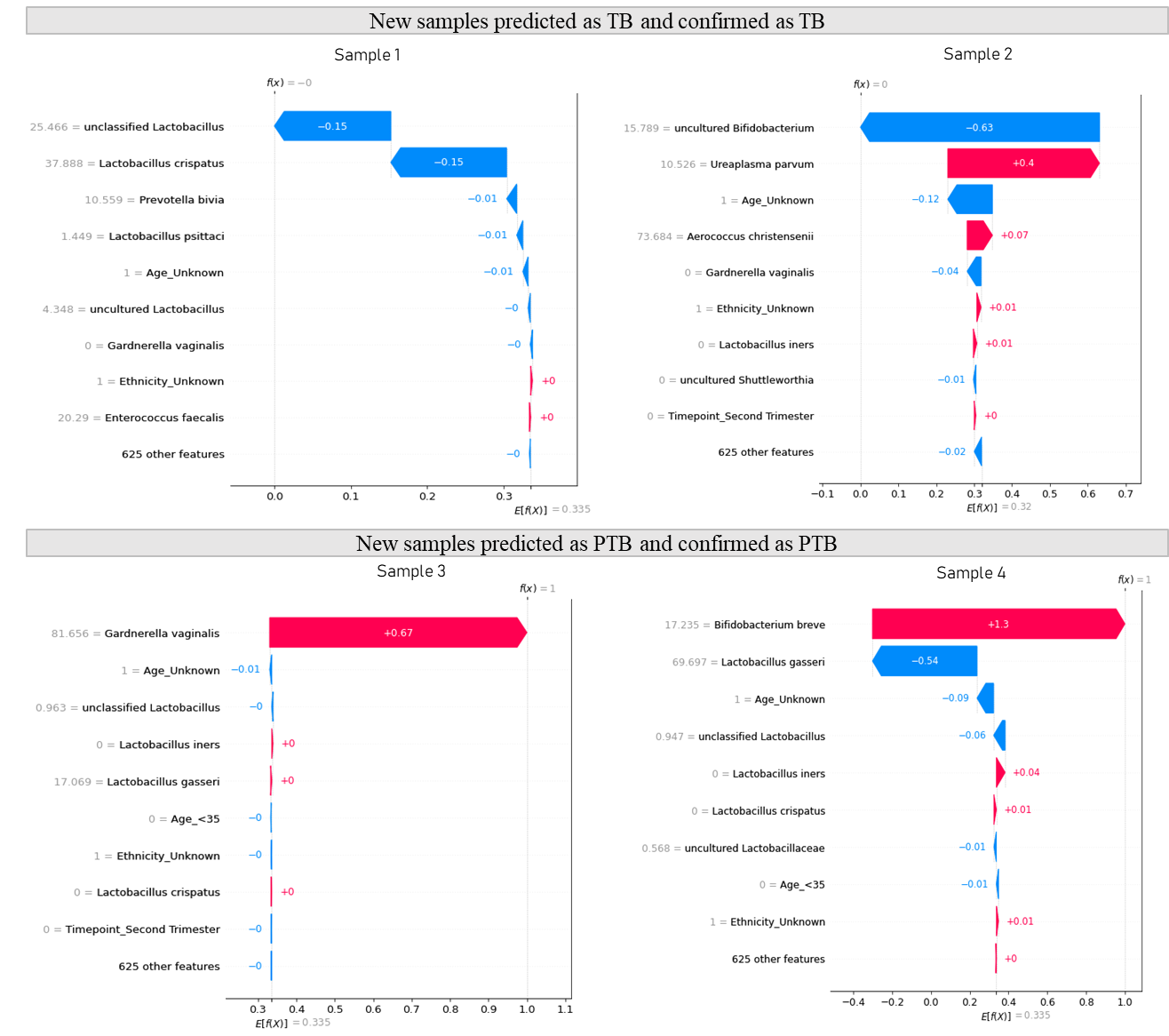
prediction were also generated using matplotlib to explain the different predictions (Fig. S6).

### Fig S6. SHAP waterfall plots obtained after phenotype prediction of 4 samples (not used in the training). The top plots refer to TB prediction on TB samples, bottom plots refer to PTB prediction on PTB samples. At the bottom of the plot, E(f(x)) corresponds to the average predicted score of the dataset. At the top of the plot, f(x) corresponds to the predicted score (0 for TB and 1 for PTB). Between these two values, the waterfall plots how each feature contributes. Each row shows how the positive (red, to PTB prediction) or negative (blue, to TB prediction) contribution of each feature changes the prediction from E(f(x)) to f(x). Features values (relative abundance for microbial species or one-hot encoded value for metadata) are indicated in grey in front of each feature.

### Computational requirements for DNN training

Supercomputing capacities (Mésocentre Clermont Auvergne University) were used for taxonomic classification. RiboTaxa took an average of 15 min per sample (20 CPU et 30 Gb RA). Due to the relatively low number of input features, model training was carried out on an i86Linux32 computer with 4.0 GB RAM × 8 cores (32.8 Gb total) without a GPU and was completed in 5 min 33 s (using complete dataset). Using the trained model, prediction of a new sample can be performed in less than 1 minute.

1. Feehily C, Crosby D, Walsh CJ, Lawton EM, Higgins S, McAuliffe FM, et al. Shotgun sequencing of the vaginal microbiome reveals both a species and functional potential signature of preterm birth. NPJ Biofilms Microbiomes. 2020;6:50.
2. Tortelli BA, Lewis AL, Fay JC. The structure and diversity of strain-level variation in vaginal bacteria. Microb Genomics. 2021;7.
3. Goltsman DSA, Sun CL, Proctor DM, DiGiulio DB, Robaczewska A, Thomas BC, et al. Metagenomic analysis with strain-level resolution reveals fine-scale variation in the human pregnancy microbiome. Genome Res. 2018;28:1467–80.
4. Fettweis JM, Serrano MG, Brooks JP, Edwards DJ, Girerd PH, Parikh HI, et al. The vaginal microbiome and preterm birth. Nat Med. 2019;25:1012–21.
5. Pace RM, Chu DM, Prince AL, Ma J, Seferovic MD, Aagaard KM. Complex species and strain ecology of the vaginal microbiome from pregnancy to postpartum and association with preterm birth. Med. 2021;2:1027-1049.e7.
6. Chakoory O, Comtet-Marre S, Peyret P. RiboTaxa: combined approaches for rRNA genes taxonomic resolution down to the species level from metagenomics data revealing novelties. NAR Genomics Bioinforma. 2022;4:lqac070.
7. Quast C, Pruesse E, Yilmaz P, Gerken J, Schweer T, Yarza P, et al. The SILVA ribosomal RNA gene database project: improved data processing and web-based tools. Nucleic Acids Res. 2013;41:D590–6.
8. Pedregosa F, Varoquaux G, Gramfort A, Michel V, Thirion B, Grisel O, et al. Scikit-learn: Machine Learning in Python. Mach Learn PYTHON.
9. Singh D, Singh B. Investigating the impact of data normalization on classification performance. Appl Soft Comput. 2020;97:105524.
10. Abadi M, Barham P, Chen J, Chen Z, Davis A, Dean J, et al. TensorFlow: a system for large-scale machine learning. Proc 12th USENIX Conf Oper Syst Des Implement. USA: USENIX Association; 2016. p. 265–83.
11. Keras: Deep Learning for humans [Internet]. Keras; 2023 [cited 2023 Mar 2]. Available from: <https://github.com/keras-team/keras>
12. Agarap AF. Deep Learning using Rectified Linear Units (ReLU) [Internet]. arXiv; 2019 [cited 2023 Mar 2]. Available from: <http://arxiv.org/abs/1803.08375>
13. Rasamoelina AD, Adjailia F, Sincak P. A Review of Activation Function for Artificial Neural Network. 2020 IEEE 18th World Symp Appl Mach Intell Inform SAMI [Internet]. Herlany, Slovakia: IEEE; 2020 [cited 2023 Jun 18]. p. 281–6. Available from: <https://ieeexplore.ieee.org/document/9108717/>
14. Abdelhafiz D, Yang C, Ammar R, Nabavi S. Deep convolutional neural networks for mammography: advances, challenges and applications. BMC Bioinformatics. 2019;20:281.
15. Khan SH, Hayat M, Porikli F. Regularization of deep neural networks with spectral dropout. Neural Netw. 2019;110:82–90.
16. Poernomo A, Kang D-K. Biased Dropout and Crossmap Dropout: Learning towards effective Dropout regularization in convolutional neural network. Neural Netw. 2018;104:60–7.
17. Glorot X, Bordes A, Bengio Y. Deep Sparse Rectifier Neural Networks. 2011 [cited 2023 Jun 11]. Available from: <https://www.semanticscholar.org/paper/Deep-Sparse-Rectifier-Neural-Networks-Glorot-Bordes/67107f78a84bdb2411053cb54e94fa226eea6d8e>
18. Kingma DP, Ba J. Adam: A Method for Stochastic Optimization [Internet]. arXiv; 2017 [cited 2023 Mar 31]. Available from: <http://arxiv.org/abs/1412.6980>
19. Müller D, Soto-Rey I, Kramer F. Multi-Disease Detection in Retinal Imaging Based on Ensembling Heterogeneous Deep Learning Models. Stud Health Technol Inform. 2021;283:23–31.
20. Rokach L, Maimon O. Decision Trees. In: Maimon O, Rokach L, editors. Data Min Knowl Discov Handb [Internet]. Boston, MA: Springer US; 2005 [cited 2023 Mar 2]. p. 165–92. Available from: <https://doi.org/10.1007/0-387-25465-X_9>
21. Mucherino A, Papajorgji PJ, Pardalos PM. k-Nearest Neighbor Classification. In: Mucherino A, Papajorgji PJ, Pardalos PM, editors. Data Min Agric [Internet]. New York, NY: Springer; 2009 [cited 2023 Mar 2]. p. 83–106. Available from: <https://doi.org/10.1007/978-0-387-88615-2_4>
22. Breiman L. Random Forests. Mach Learn. 2001;45:5–32.
23. Webb GI. Naïve Bayes. In: Sammut C, Webb GI, editors. Encycl Mach Learn [Internet]. Boston, MA: Springer US; 2010 [cited 2023 Mar 2]. p. 713–4. Available from: <https://doi.org/10.1007/978-0-387-30164-8_576>
24. Chen T, Guestrin C. XGBoost: A Scalable Tree Boosting System. Proc 22nd ACM SIGKDD Int Conf Knowl Discov Data Min. 2016;785–94.
25. Cox DR. The Regression Analysis of Binary Sequences. J R Stat Soc Ser B Methodol. 1958;20:215–32.
26. Cortes C, Vapnik V. Support-vector networks. Mach Learn. 1995;20:273–97.
27. Adnan M, Alarood AAS, Uddin MI, ur Rehman I. Utilizing grid search cross-validation with adaptive boosting for augmenting performance of machine learning models. PeerJ Comput Sci. 2022;8:e803.
28. Truong DT, Franzosa EA, Tickle TL, Scholz M, Weingart G, Pasolli E, et al. MetaPhlAn2 for enhanced metagenomic taxonomic profiling. Nat Methods. 2015;12:902–3.
29. Segata N, Waldron L, Ballarini A, Narasimhan V, Jousson O, Huttenhower C. Metagenomic microbial community profiling using unique clade-specific marker genes. Nat Methods. 2012;9:811–4.
30. Liang Q, Bible PW, Liu Y, Zou B, Wei L. DeepMicrobes: taxonomic classification for metagenomics with deep learning. NAR Genomics Bioinforma [Internet]. 2020 [cited 2021 Jan 12];2. Available from: <https://doi.org/10.1093/nargab/lqaa009>
31. Huang W, Li L, Myers JR, Marth GT. ART: a next-generation sequencing read simulator. Bioinformatics. 2012;28:593–4.
32. Hajian-Tilaki K. Receiver Operating Characteristic (ROC) Curve Analysis for Medical Diagnostic Test Evaluation. Casp J Intern Med. 2013;4:627–35.
33. Hunter JD. Matplotlib: A 2D Graphics Environment. Comput Sci Eng. 2007;9:90–5.
34. Plotly: Low-Code Data App Development [Internet]. [cited 2023 Jul 3]. Available from: <https://plotly.com/>
35. Efron B, Tibshirani RJ. An Introduction to the Bootstrap. New York: Chapman and Hall/CRC; 1994.
36. DeLong ER, DeLong DM, Clarke-Pearson DL. Comparing the areas under two or more correlated receiver operating characteristic curves: a nonparametric approach. Biometrics. 1988;44:837–45.
37. Saarela M, Jauhiainen S. Comparison of feature importance measures as explanations for classification models. SN Appl Sci. 2021;3:272.
38. Lundberg S, Lee S-I. A Unified Approach to Interpreting Model Predictions [Internet]. arXiv; 2017 [cited 2023 Mar 2]. Available from: <http://arxiv.org/abs/1705.07874>
39. Shrikumar A, Greenside P, Kundaje A. Learning Important Features Through Propagating Activation Differences [Internet]. arXiv; 2019 [cited 2023 Mar 30]. Available from: <http://arxiv.org/abs/1704.02685>
40. Baud A, Hillion K-H, Plainvert C, Tessier V, Tazi A, Mandelbrot L, et al. Microbial diversity in the vaginal microbiota and its link to pregnancy outcomes. Sci Rep. 2023;13:9061.
